# Supplementary material for: Causal Relationship between Aging and Anorexia Nervosa: A White-Matter-Microstructure-Mediated Mendelian Randomization Analysis
Source: Biomedicines. 2024 Aug 16;12(8):1874. doi: 10.3390/biomedicines12081874 (PMC11351342; doi:10.3390/biomedicines12081874)
Supplement: Supplementary file 1 [file biomedicines-12-01874-s001.zip › Supplementary Figure/Figure S4/Singe SNP plot of MR analysis for TL to ubm-b-1555.pdf]

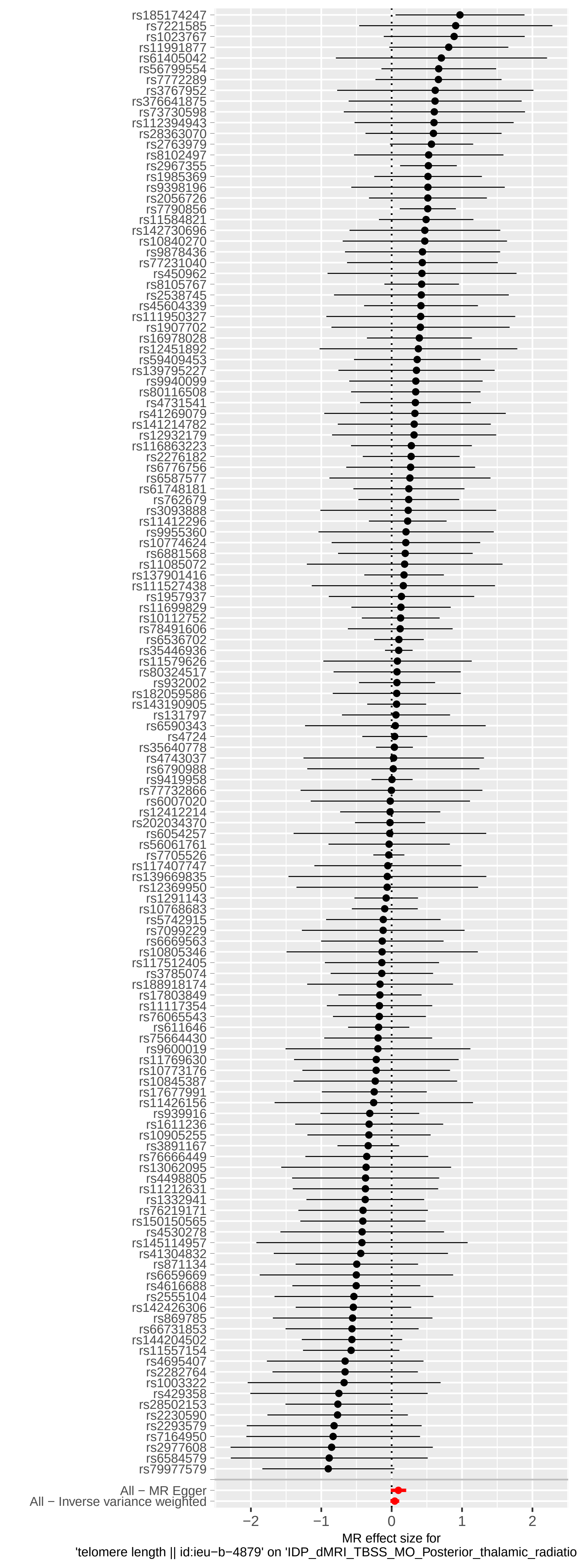

rs185174247
rs7221585
rs1023767
rs11991877
rs61405042
rs56799554
rs7772289
rs3767952
rs376641875
rs73730598
rs112394943
rs28363070
rs2763979
rs8102497
rs2967355
rs1985369
rs9398196
rs2056726
rs7790856
rs11584821
rs142730696
rs10840270
rs9878436
rs77231040
rs450962
rs8105767
rs2538745
rs45604339
rs111950327
rs1907702
rs16978028
rs12451892
rs59409453
rs139795227
rs9940099
rs80116508
rs4731541
rs41269079
rs141214782
rs12932179
rs116863223
rs2276182
rs6776756
rs6587577
rs61748181
rs762679
rs3093888
rs11412296
rs9955360
rs10774624
rs6881568
rs11085072
rs137901416
rs111527438
rs1957937
rs11699829
rs10112752
rs78491606
rs6536702
rs35446936
rs11579626
rs80324517
rs932002
rs182059586
rs143190905
rs131797
rs6590343
rs4724
rs35640778
rs4743037
rs6790988
rs9419958
rs77732866
rs6007020
rs12412214
rs202034370
rs6054257
rs56061761
rs7705526
rs117407747
rs139669835
rs12369950
rs1291143
rs10768683
rs5742915
rs7099229
rs6669563
rs10805346
rs117512405
rs3785074
rs188918174
rs17803849
rs11117354
rs76065543
rs611646
rs75664430
rs9600019
rs11769630
rs10773176
rs10845387
rs17677991
rs11426156
rs939916
rs1611236
rs10905255
rs3891167
rs76666449
rs13062095
rs4498805
rs11212631
rs1332941
rs76219171
rs150150565
rs4530278
rs145114957
rs41304832
rs871134
rs6659669
rs4616688
rs2555104
rs142426306
rs869785
rs66731853
rs144204502
rs11557154
rs4695407
rs2282764
rs1003322
rs429358
rs28502153
rs2230590
rs2293579
rs7164950
rs2977608
rs6584579
rs79977579
All − MR Egger
All − Inverse variance weighted
−2
−1
0
1
2
MR effect size for
'telomere length || id:ieu−b−4879' on 'IDP_dMRI_TBSS_MO_Posterior_thalamic_radiatio
